# Optimal utilization of mechanical circulatory support and transplant resources in the comprehensive treatment of terminal heart failure

**DOI:** 10.1186/1749-8090-8-S1-P105

**Published:** 2013-09-11

**Authors:** L Svetina, M Petricevic, B Biocina

**Affiliations:** 1 Department of Cardiac Surgery, University Hospital Centre Zagreb, Zagreb, Croatia

## Background

Treatment of end-stage heart failure has markedly evolved. With only a limited number of available heart transplants (24 in 2012) in our institution, mechanical circulatory support (MCS) has become an integral part of acute and end-stage heart failure treatment and has improved survival. We report our experience in MCS program since 2008.

## Methods

Short, intermediate and long term MCS can be instituted for different clinical indications, ranging from postcardiotomy circulatory failure, acute cardiogenic or respiratory shock, chronic heart failure in patients not eligible for a transplant to heart transplant function deterioration. From September 2008 to June 2013, 94 patients were eligible for MCS. The majority of MCS patients were adults, 89/94. The adult group of 89 patients underwent 110 MCS procedures, with 16 patients experiencing multiple procedures.

## Results

In the adult group, 66% of patients were at INTERMACS level 1, critical cardiogenic shock, and 19% were at level 2, progressive decline. Indications for primary MCS included: acute exacerbation of chronic heart failure, acute cardiogenic shock and respiratory failure. Altogether, procedural success was accomplished in 49.4% of patients. MCS efficiently bridged 14 patients with heart failure to heart transplantation. In 15 patients, long term support was instituted, either as a destination therapy or as a bridge to heart transplant; 13 received HeartMate II, and 2 patients received HeartWare. One patient with respiratory failure was successfully bridged to lung transplantation. In the pediatric group, 60% patients were bridged to recovery. Postcardiotomy MCS was used in 33.7% patients. Patient outcome after 30 days was assessed as: alive (22.4%), alive on support (13.5%), dead (64.0%). The most common perioperative complication was renal failure, in 42.7% patients.

## Conclusion

MCS has become an integral part of terminal stage heart failure treatment alongside established standard procedures.

